# Proof-of-Concept Study of Multifunctional Hybrid Nanoparticle System Combined with NIR Laser Irradiation for the Treatment of Melanoma

**DOI:** 10.3390/biom11040511

**Published:** 2021-03-30

**Authors:** Joana Lopes, Tânia Ferreira-Gonçalves, Isabel V. Figueiredo, Cecília M. P. Rodrigues, Hugo Ferreira, David Ferreira, Ana S. Viana, Pedro Faísca, Maria Manuela Gaspar, João M. P. Coelho, Catarina Oliveira Silva, Catarina Pinto Reis

**Affiliations:** 1 Research Institute for Medicines (iMed.ULisboa), Faculty of Pharmacy, Universidade de Lisboa, Av. Professor Gama Pinto, 1649-003 Lisboa, Portugal; joanamargaridalopes@campus.ul.pt (J.L.); taniag1@ff.ulisboa.pt (T.F.-G.); cmprodrigues@ff.ulisboa.pt (C.M.P.R.); mgaspar@ff.ulisboa.pt (M.M.G.); catarina.m.oliveira.silva@gmail.com (C.O.S.); 2 Pharmacology and Pharmaceutical Care Laboratory, Faculty of Pharmacy, University of Coimbra, Azinhaga de Santa Comba, 3000-548 Coimbra, Portugal; isabel.vitoria@netcabo.pt; 3 Institute for Clinical and Biomedical Research (iCBR), Faculty of Medicine, University of Coimbra, Azinhaga de Santa Comba, 3000-548 Coimbra, Portugal; 4 Instituto de Biofísica e Engenharia Biomédica, Faculdade de Ciências, Campo Grande, Universidade de Lisboa, 1749-016 Lisboa, Portugal; hugoferreira@campus.ul.pt (H.F.); jmcoelho@fc.ul.pt (J.M.P.C.); 5 MED-Mediterranean Institute for Agriculture, Environment and Development, Department of Veterinary Medicine, University of Évora, Pólo da Mitra, 7002-554 Évora, Portugal; ferreiradav@gmail.com; 6 Centro de Química Estrutural, Faculdade de Ciências, Universidade de Lisboa, 1749-016 Lisboa, Portugal; apsemedo@fc.ul.pt; 7 CBIOS-Research Center for Biosciences & Health Technologies, Universidade Lusófona de Humanidades e Tecnologias, Campo Grande 376, 1749-024 Lisboa, Portugal; pedrofaisca76@gmail.com; 8 Faculty of Veterinary Medicine, Universidade Lusófona de Humanidades e Tecnologias, Campo Grande 376, 1749-024 Lisboa, Portugal; 9 Department of Biomedical Sciences, Faculty of Pharmacy, Campus Universitario, University of Alcalá, Ctra. A2 km 33,600, 28871 Alcalá de Henares, Spain

**Keywords:** multifunctional nanoparticles, gold nanoparticles, targeted therapy, photothermal therapy, superficial tumors, melanoma, experimental models

## Abstract

The global impact of cancer emphasizes the importance of developing innovative, effective and minimally invasive therapies. In the context of superficial cancers, the development of a multifunctional nanoparticle-based system and its in vitro and in vivo safety and efficacy characterization are, herein, proposed as a proof-of-concept. This multifunctional system consists of gold nanoparticles coated with hyaluronic and oleic acids, and functionalized with epidermal growth factor for greater specificity towards cutaneous melanoma cells. This nanoparticle system is activated by a near-infrared laser. The characterization of this nanoparticle system included several phases, with in vitro assays being firstly performed to assess the safety of gold nanoparticles without laser irradiation. Then, hairless immunocompromised mice were selected for a xenograft model upon inoculation of A375 human melanoma cells. Treatment with near-infrared laser irradiation for five minutes combined with in situ administration of the nanoparticles showed a tumor volume reduction of approximately 80% and, in some cases, led to the formation of several necrotic foci, observed histologically. No significant skin erythema at the irradiation zone was verified, nor other harmful effects on the excised organs. In conclusion, these assays suggest that this system is safe and shows promising results for the treatment of superficial melanoma.

## 1. Introduction

Cancer is a worldwide scourge and one of the most feared diseases. Over the last few decades, its incidence has been increasing, probably as a consequence of lifestyle changes, as well as increased life expectancy [1,2]. Melanoma, in turn, is a complex malignant tumor originated from melanocytes, and it is generally located at the skin level (i.e., cutaneous melanoma) although, in a smaller percentage, it can also develop in the mucosa, retina and meninges (i.e., non-cutaneous melanoma) [3]. Despite presenting a low incidence, melanoma is reported as the most aggressive type of skin tumor, with a high associated mortality rate each year [1,3,4].

Although several differentiated approaches have been used to treat melanoma over the last years, increased tumor-targeted properties, better efficacy profiles, and less side effects are essential to improve the outcomes [5,6]. In this segment, photothermal therapy (PTT) emerges as a highly promising strategy, once it implies minimal invasion in comparison to other currently available therapies, presents a safe profile, and allows a faster patient recovery [7]. Briefly, PTT is a precise spatial-temporal technique causing local hyperthermia with tissues temperature ranging from 41 to 47 °C [8,9] in tumor cells after irradiation with light beams [10,11]. This therapy is mostly studied for treatment of superficial tumors due to the light limited ability of deeply penetrating into the tissues. An option to increase the therapeutic value of this technique includes the use of near-infrared (NIR) radiation, which is less absorbed by the tissues, allowing to achieve higher tissue depth during tumor treatment [11,12].

The use of gold nanoparticles (GNPs) in PTT have demonstrated promising results as photothermal enhancers, due to their plasmonic tunable properties [13] and surface functionalization [14,15]. When administered directly into the tumor (i.e., intra-tumoral injection), GNPs show higher accumulation and tumor residence compared to systemic routes (e.g., tail vein intravenous (i.v.) injection) [16]. The addition of targeting moieties and ligands to GNPs by physical adsorption of coating polymers or linkers, also promotes a more localized and selective action [17,18,19].

To evaluate the toxicity and efficiency of antitumoral therapies, pre-clinical studies still depend greatly on animal models [20,21]. Several cancer models have been developed [22,23] being murine models the most frequently used [24]. Moreover, xenograft models are widely preferred, as they allow the transplantation of human cancer cells into mice [25,26]. Human melanoma xenograft models show advantages over other models, such as the possibility to control tumor growth and experimental points, by injecting cells through a standardized technique [27], and the ability to mimic the histological and genetic characteristics of the original tumors [21]. Further, there is a vast list of well-established cell lines characterized from different tumor types for these animal models [26,28,29]. The A375 human melanoma cell line is most commonly used in xenograft models, due to its successful implementation [29,30,31,32], both for local orthotopic tumor development through intra-dermal (i.d.) administration [33], and metastases (e.g., lungs and brain), when administered i.v. [34].

This study reports a proof-of-concept for assessing the in vitro and in vivo toxicity, and efficacy profile of a previously developed and partly characterized multifunctional hybrid nanoparticle system, developed for NIR-mediated PTT [15,35], in mice induced melanoma model upon inoculation with A375 human melanoma cells, which overexpress multiple receptors [36,37], including CD44 and epidermal growth factor (EGF) receptors [38,39,40,41]. This gold nanoparticle-based system coated with hyaluronic (HA) and oleic acids (OA), includes EGF as ligand, EGF-conjugated HAOA-coated GNPs. The coupling of HA and EGF at nanoparticle surface aimed to improve the in vivo efficiency of the GNPs, by specifically targeting CD44 [42] and EGF [40] receptors, respectively.

These in vitro and in vivo assays were performed in three phases: (1) safety evaluation of the GNP formulation; (2) safety evaluation of laser exposure in severe combined immune-deficient (SCID) hairless mice model; and (3) efficacy evaluation of the multifunctional hybrid system in melanoma tumors inoculated in SCID hairless mice by comparing the effect of using the laser irradiation alone or combined with the in situ injection of hybrid nanoparticles.

## 2. Materials and Methods

### 2.1. Materials

Gold (III) chloride trihydrate (HAuCl_4_·3H_2_O), hyaluronic acid (HA) sodium salt from *Streptococcus equi* (MW 7000–250,000 g/mol), oleic acid (OA) (MW 282.46 g/mol), trypsin, fetal bovine serum (FBS), and penicillin/streptomycin were all supplied from Sigma-Aldrich (Steinheim, Germany). Dulbecco’s modified Eagle medium (DMEM) was supplied by Biowest (Nuaillé, France). The human keratinocyte cell line, HaCat, the human melanoma cell line, A375, and the murine melanoma cell line, B16F10, were provided by Cell Line Service GmbH (Eppelheim, Germany). Recombinant human epidermal growth factor (EGF) was purchased from Life Technologies (Waltham, MA, USA). The water used in all the experiments was purified through a Millipore system (Millipore, Burlington, MA, USA). All the remaining chemicals and substrates used were of analytical grade.

Intramuscular administration of a mixture of ketamine (Ketamidor^®^, Richter Pharma, Wels, Austria) and chlorpromazine (Largactil^®^, Laboratórios Vitória, S.A., Amadora, Portugal) (10:2 *v*/*v*) at a dose of 2 mL/kg was used as anesthetic protocol for the in vivo experiments.

### 2.2. Preparation and Characterization of EGF-Conjugated HAOA-Coated GNPs

The multifunctional hybrid system, EGF-conjugated HAOA-coated GNPs, was prepared as previously and fully described [15,35,43]. Briefly, uncoated GNPs were produced based on the addition of an aqueous solution containing ascorbic acid, silver nitrate, and rosmarinic acid, all of them acting as reducing agents of the gold salt, in substitution of toxic chemicals commonly used like cetyl trimethylammonium bromide (CTAB). Then, the uncoated GNPs reacted with the coating mixture of HA and OA with a final concentration of 0.5 mg/mL. Later, EGF peptide (10 µg/mL) was added to the formulation and allowed to interact with HAOA-coated GNPs for 30 min under magnetic stirring (at 800 rpm). EGF-conjugated HAOA-coated GNPs were stored for 24 h at 4 °C, protected from light, and afterwards centrifuged at 10,900× *g* for 10 min (Hermle Labortechnik Gmbh, Wehingen, Germany) to remove unbound peptides. Finally, EGF-conjugated HAOA-coated GNPs were frozen, lyophilized for 24 h at −50.0 ± 2.0 °C, in FreeZone 2.5 L Benchtop Freeze Dry System (Labconco, Kansas City, MO, USA) and stored at −20 °C until the day of the experiments.

Subsequently, uncoated, HAOA-coated and EGF-conjugated HAOA-coated GNPs were characterized in terms of mean particle size and polydispersity index (PdI) (samples diluted in water, 2:10) through dynamic light scattering (DLS) (Zetasizer Nano S; Malvern Instruments, Malvern, UK), and zeta potential (samples diluted in phosphate buffer saline (PBS), according USP30 and at pH 7.4, 2:10) by electrophoretic mobility technique (Zetasizer Nano Z; Malvern Instruments, Malvern, UK), in 3 series of 11 measurements of each analyzed sample.

The morphology of GNPs was evaluated by atomic force microscopy (AFM) analysis. Succinctly, 40 μL of sample were placed on a freshly cleaved mica surface and allowed to dry overnight before analysis. Images were acquired by Multimode 8 HR coupled to Nanoscope V Controller (Bruker, Coventry, UK), using peak force tapping and ScanAssist mode. Tip model used was scanasyst-air 0.4 N/m, Bruker.

The absorbance spectra of the EGF-conjugated HAOA-coated GNPs were also obtained by UV–visible spectroscopy (Shimadzu UV-160A; Shimadzu Europa GmbH, Duisburg, Germany).

### 2.3. In Vitro Safety Assays on HaCat, A375, and B16F10 Cell Lines

The cytotoxicity of uncoated, HAOA-coated and EGF-conjugated HAOA-coated GNPs was evaluated in three cell lines: healthy human keratinocytes (HaCat), human melanoma cells (A375) and murine melanoma cells (B16F10). These commercial cell lines are well established, widely used and characterized in the literature, namely, in terms of membrane receptors. HaCat [44] and A375 [45] cells are known for their high expression of EGFR while, in contrast, B16F10 cells express low levels of this receptor [46]. Furthermore, the three cell lines have been associated with the presence of CD44 membrane receptors [47], with the lowest expression in HaCat cells and the highest in B16F10 cells. Cell lines were grown in DMEM with glucose (4500 mg/L), supplemented with 10% FBS and 100 IU/mL penicillin and 100 μg/mL streptomycin (Invitrogen; complete medium). Cells were preserved at 37 °C under a 5% CO_2_ atmosphere and cultures were checked and maintained every 2–3 days, until achieving a confluence of about 80%.

Primarily, the cell lines were incubated with increasing concentrations of uncoated and HAOA-coated GNPs (25, 50, 75, and 100 μM). These concentrations were based on the content of gold. Afterwards, the cytotoxicity of the final formulation, i.e., EGF-conjugated HAOA-coated GNPs, was evaluated at the maximum concentration tested in previous assays. The incubation period was 24 h for all assays.

The cell viability was evaluated using the 3-(4,5-dimethylthiazol-2-yl)-2,5-diphenyltetrazolium bromide (MTT) assay [48]. Succinctly, cells were seeded in 96-well plates (200 μL) at 5 × 10^4^ cells/mL and allowed to adhere, overnight, in the culture conditions specified above. Thereafter, the complete medium was removed, and the cells were incubated at 37 °C under a 5% CO_2_ atmosphere for 24 h with GNPs. After the incubation period, medium was discarded, and cells were washed two times with PBS. Afterwards, 50 μL of MTT at a concentration of 0.5 mg/mL in incomplete medium were added to each well, and plates were incubated for 4 h at 37 °C under a 5% CO_2_ atmosphere. Then, to solubilize the formazan crystals, 100 μL of dimethyl sulfoxide (DMSO) were added to each well and plates were shaken for 10–15 min. Finally, absorbance was measured in a microplate reader at 570 nm (BioTek ELx800; BioTek Instruments, Inc., Winooski, VT, USA). The cell viability was evaluated by determining the percentage of viable cells ($cell\;viability\;\left(\%\right)=\frac{ODt}{ODc}\times 100,$ where ODt is the optical density of the cells incubated with the tested formulations and ODc is the optical density of the control cells, corresponding to 100% cell viability).

In addition to the evaluation of cytotoxicity, the presence of the GNPs in A375 cells, the same ones used in the xenograft model reported in the following section, was addressed by atomic force and optical microscopy. Experimental details are presented in Supplementary Materials.

### 2.4. Human Melanoma Xenograft Model

This study was conducted using immunosuppressed mice for T and B cells, and human melanoma A375 cell line (ATCC^®^ CRL-1619™), in the animal facilities of the Faculty of Pharmacy, University of Coimbra.

Hairless 42 days-old male SCID mice (SHO^®^, SCID Hairless Outbred, code: 474, Charles River, Barcelona, Spain) were selected for the establishment of melanoma xenografts models. This animal model tends to reduce formation of metastases and delay cell growth, as demonstrated for the transformed A375 cell line with lung tropism due to the presence of natural killer (NK) cells [29]. Animals were allowed to adapt to the laboratory conditions for 7 days before testing, maintained with food and water ad libitum and kept at 22.0 ± 1.0 °C with controlled 12 h light/dark cycle. After this period, the cells were inoculated in the fold-back of the animal neck at a concentration of 1 × 10^6^ A375 cells/ mouse in 200 µL of PBS pH 7.4, as described in the literature [49].

Cells were cultured according to the previous section and, at the day of the injection (tumor induction day), were treated with a Versene solution (Gibco^®^, Thermofisher, New York, NY, USA) and NaCO_3_ (Gibco^®^, Thermofisher, New York, USA) for alkalinization, and were harvested with trypsin and ethylenediaminetetraacetic acid (EDTA). Then, cells were centrifuged at 800× *g* for 5 min (Eppendorf AG, Hamburg, Germany) and suspended in PBS at pH 7.4 for posterior injection in mice.

The animals were monitored twice a week for weight control, body conditions (body condition score—BCS), behavior and signs of tumor progression. The size of the tumors and rate of growth were measured using a caliper, until reaching palpable tumor size. A minimum tumor volume of approximately 5–10 mm^3^ was required for inclusion in the study. Tumor volume was calculated considering that $tumor\;volume=\frac{length\times \;width^{2}}{2}$, where length is the largest diameter and width is the smallest diameter perpendicular to the length.

### 2.5. Photothermal Therapy

#### 2.5.1. Laser Irradiation Procedure and Treatment Evaluation in SCID Mice

**Figure 1:**
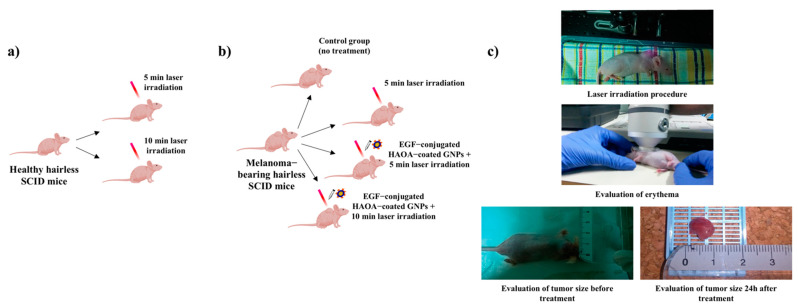
Illustrative representation of the different groups created to assess: (**a**) the safety of the laser exposure; and (**b**) the anti-cancer efficacy of the treatments. (**c**) Experimental setup.

In order to assess the safety of the laser exposure alone, at an initial phase, and the efficacy of the treatment with laser irradiation combined with the EGF-conjugated HAOA-coated GNPs, different groups of healthy and melanoma-bearing SCID hairless mice were created (Figure 1). The animals were divided as follows: (i) for safety evaluation of the laser exposure, six mice without tumors were randomly distributed in two groups: (1) exposed to 5 min laser irradiation (*n* = 3), and (2) exposed to 10 min laser irradiation (*n* = 3); (ii) for evaluation of the efficacy of different treatments, mice with palpable tumors were randomly assigned to: (1) control group (i.e., no treatment) (*n* = 3); (2) 5 min laser irradiation (*n* = 3); (3) intra-tumoral injection of EGF-conjugated HAOA-coated GNPs followed by exposure to the 5 min laser irradiation (*n* = 4); and (4) intra-tumoral injection of EGF-conjugated HAOA-coated GNPs followed by 10 min laser irradiation (*n* = 3). In Groups 3 and 4, EGF-conjugated HAOA-coated GNPs were reconstituted in PBS at the time of administration and injected at the tumor site (i.t.) at a concentration, per mouse, of 20 mg/kg of nanoparticles in 100 µL. The GNPs dose was selected based on gold concentration, which corresponds to about 1.25 mg/kg. Moreover, it considered the in vitro data obtained here and in previous works [15,35], where concentrations up to 80 µM of gold did not show significant toxicity.

The NIR laser (JDSU L4-2495-003 coupled to a source Laserpak ARO-485-08-05) used in these experiments had a wavelength of 811 nm and 2.5 W/cm^2^ irradiance at the target. The time of exposure was chosen based on our previous in vitro results [50] and considered exposure times and irradiation doses found in the literature [51,52] as well as the exposure limits in guidelines for the use of NIR radiation [53,54].

All animals exposed to laser irradiation were previously anesthetized, which in the case of the animals also treated with GNPs occurred 4 h after the injection with EGF-conjugated HAOA-coated GNPs. This 4 h time frame was adopted based on the cell internalization time necessary for the same type of GNPs, previously reported by our group [35]. Moreover, additional studies to evaluate the presence of the GNPs in the A375 cell line were carried out and revealed the presence of these particles upon 4 h of incubation (See Figures S1 and S2). The laser beam was applied in the center of the tumors. The animals were continuously monitored during the irradiation time (5 or 10 min), and until recovering the righting reflex. Photographs and body weights were also registered before and after laser irradiation procedure.

#### 2.5.2. In Vivo Evaluation of Safety and Efficacy from the Photothermal Therapy Procedure

During irradiation, animals were closely monitored to ensure that no burns were induced. Furthermore, before and immediately after laser exposure, mice were evaluated for possible formation of erythema, at the irradiation zone, by colorimetry (a*, AU) (Minolta Chroma meter CR-300; Konica Minolta Sensing Americas, Inc., Ramsey, NJ, USA).

At 24 h post treatment, all animals were sacrificed, according to the applicable animal welfare principles. The size and volume of the tumors were registered before and 24 h after treatment. Of notice, while the size of the tumors before the treatments was measured still in the animal, accounting also the skin and other tissues surrounding the tumor, the tumor size after treatments was measured after their excision, accounting only the tumor mass.

In addition to tumors, internal organs (i.e., lungs, kidneys, liver, spleen, and heart) were also excised, weighted, measured, and prepared for histological evaluation. The tissue index for each organ was calculated based on the organ and animal weight by applying the equation$:\;tissue\;index=\sqrt{\frac{organ\;weight}{body\;weight}}\times 100$ [55].

The tissue for analysis was fixed in 10% formalin, paraffin embedded, and cut into five-micrometer sections for hematoxylin-eosin staining (H&E staining). Slices were examined under an Olympus BX51 microscope (Olympus Corporation, Tokyo, Japan), images were taken using an Olympus U-TV1X-2 color camera and the extent of tumor necrosis was analyzed with Olympus analySIS^®^ software (Olympus Corporation, Tokyo, Japan).

### 2.6. Statistical Analysis

The cell viability of in vitro assays was expressed as mean ± standard deviation (SD) and the statistical analysis was evaluated by two-way ANOVA followed by Tukey’s multiple comparisons test. The average weights of the groups of animals with and without tumors was described as mean ± standard error of the mean (SEM), and the values were compared through a Student’s *t*-test for independent (unpaired) samples. Differences in animal weight over time were assessed through a two-way ANOVA followed by Dunnett’s multiple comparisons test. The statistical analysis of the erythema was assessed by a one-way ANOVA followed by Dunnett’s multiple comparison test, assuming that the control group was kept unchanged for erythema (difference of 0). Lastly, tissue indexes were compared among the treatment groups using a two-way ANOVA followed by Tukey’s multiple comparisons test. P-values were presented for comparisons whenever applicable. A *p*-value < 0.05 was considered as statistically significant. All statistical analyses were performed using GraphPad Prism version 8.0.2 (GraphPad Software, San Diego, CA, USA).

## 3. Results

### 3.1. Characterization of EGF-Conjugated HAOA-Coated GNPs

**Figure 2:**
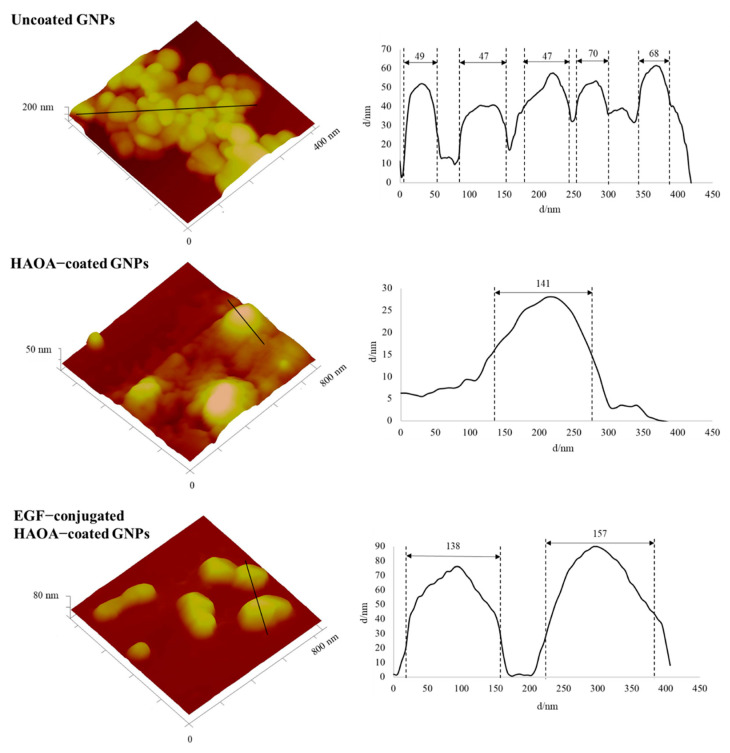
Dimensional atomic force microscopy images with corresponding cross-section profiles of uncoated GNPs, HAOA-coated GNPs and EGF-conjugated HAOA-coated GNPs

The multifunctional hybrid nanoparticles produced for these in vitro and in vivo experiments had similar characteristics to those previously reported by Silva et al. [15,35]. EGF-conjugated HAOA-coated GNPs presented spherical morphology (Figure 2) with a mean size of 157 nm (PdI ≤ 0.4) and a negative surface charge (−19 ± 9 mV) (Table 1). Moreover, the nanoparticles showed a plasmonic absorption band at the NIR range (650–900 nm), as previously observed [15].

### 3.2. In Vitro Safety Assays on HaCat, A375 and B16F10 Cell Lines

The potential cytotoxicity of uncoated, HAOA-coated and EGF-conjugated HAOA-coated GNPs was assessed on HaCat, A375 and B16F10 cells by MTT assay. This assay relies on the fact that living cells have the ability to transform MTT into formazan crystals, reflecting its mitochondrial activity. Since mitochondrial activity is constant for most cell lines, it is assumed that variations in cell viability are reflected in the amount of formazan crystals formed [56].

**Figure 3:**
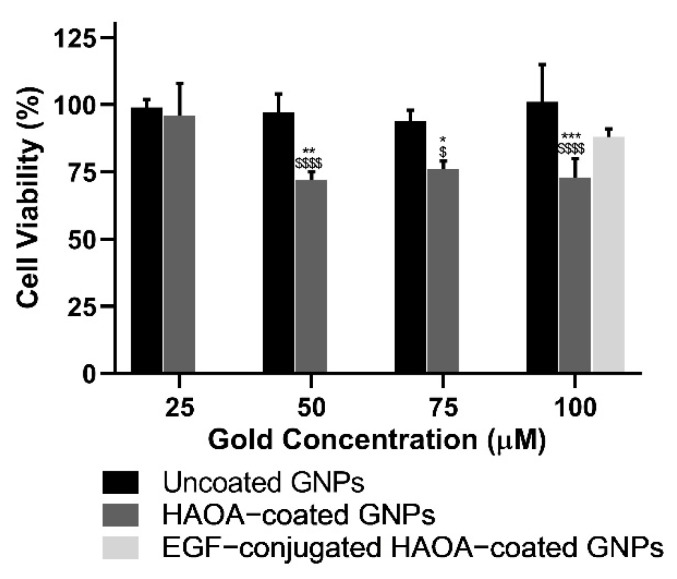
Cell viability (%) of HaCat cell line 24 h after incubation with uncoated and HAOA-coated GNPs at different concentrations, and EGF-conjugated HAOA-coated GNPs at 100 μM. * *p* < 0.05, ** *p* < 0.01 and *** *p* < 0.001 comparing to HAOA-coated GNPs at 25 µM. $ *p* < 0.05 and $$$$ *p* < 0.0001 comparing to uncoated GNPs in the same concentrations. Data are presented as mean value ± SD, *n* ≥ 3.

**Figure 4:**
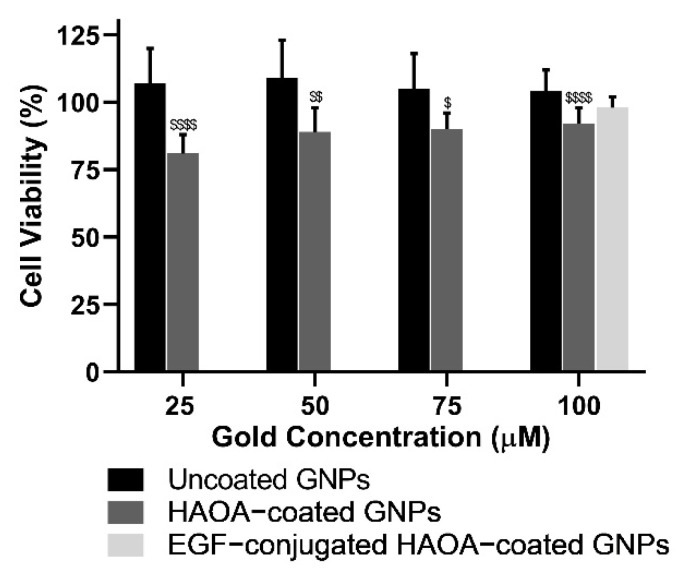
Cell viability (%) of A375 cell line 24 h after incubation with uncoated and HAOA-coated GNPs at different concentrations, and EGF-conjugated HAOA-coated GNPs at 100 μM. $ *p* < 0.05, $$ *p* < 0.01 and $$$$ *p* < 0.0001 comparing to uncoated GNPs in the same concentrations. Data are presented as mean value ± SD, *n* ≥ 3.

**Figure 5:**
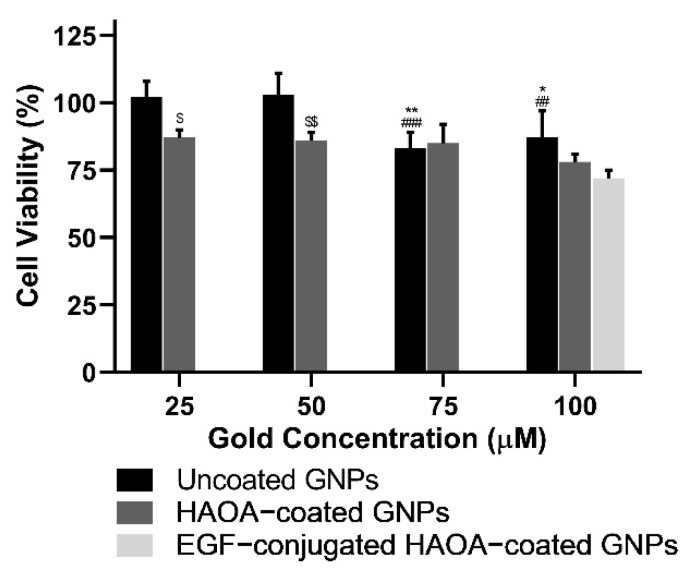
Cell viability (%) of B16F10 cell line 24 h after incubation with uncoated and HAOA-coated GNPs at different concentrations, and EGF-conjugated HAOA-coated GNPs at 100 μM. * *p* < 0.05 and ** *p* < 0.01 comparing to uncoated GNPs at 25 µM. ## *p* < 0.01 and ### *p* < 0.001 comparing to uncoated GNPs at 50 µM. $ *p* < 0.05 and $$ *p* < 0.01 comparing to uncoated GNPs in the same concentrations. Data are presented as mean value ± SD, *n* ≥ 3.

In a first phase, cell viability was evaluated in the presence of increasing gold-based concentrations of uncoated and HAOA-coated GNPs, ranging from 25 to 100 μM. Then, the cell viability of the final formulation, i.e., EGF-conjugated HAOA-coated GNPs, was also evaluated at 100 μM. Results are depicted in the following figures (Figure 3, Figure 4 and Figure 5).

Regarding healthy human keratinocytes, cell viability with uncoated GNPs was similar to that of the controls (≥94%), regardless of the concentrations tested. Despite preserving cell viability unchanged at the lowest concentration (25 μM), HAOA-coated GNPs at 50, 75, and 100 μM caused a significant reduction of about 26% (*p* < 0.05) on cell viability. Conversely, EGF-conjugated HAOA-coated GNPs at 100 μM produced a non-statistically significant changes in HaCat cell viability in comparison with uncoated and HAOA-coated GNPs at the same concentration.

With regard to the A375 cell line, similarly to what happened in HaCat cells, uncoated GNPs did not affect cell viability regardless of the concentrations tested. By its turn, for HAOA-coated GNPs a slight reduction on cells viability at all concentrations studied was observed. This decrease was statistically significant (*p* < 0.05) when compared to uncoated GNPs at each respective concentration. In addition, there was no noticeable loss of cell viability for EGF-conjugated HAOA-coated GNPs at 100 μM.

While for HaCat and A375 cells, uncoated GNPs were safe in all concentrations, for B16F10 cells a slight reduction on cell viability was attained: 17% and 13%, when comparing to control cells at the higher concentrations tested, 75 and 100 µM, respectively. HAOA-coated GNPs slightly affected B16F10 cell viability but, in this case, for all tested concentrations. By its turn, for EGF-conjugated HAOA-coated GNPs at 100 μM, a reduction in B16F10 cell viability of approximately 28% was exhibited.

**Figure 6:**
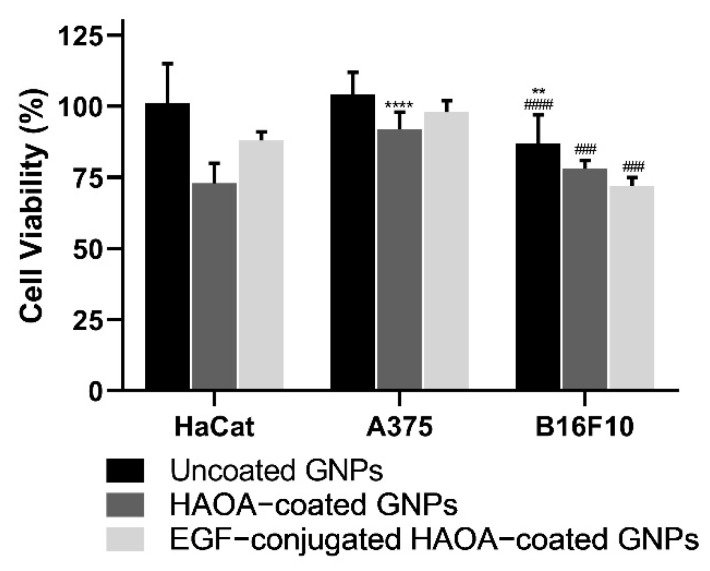
Comparative cell viability (%) of HaCat, A375 and B16F10 24 h after incubation with uncoated, HAOA-coated GNPs and EGF-conjugated HAOA-coated GNPs at 100 μM. ** *p* < 0.01 and **** *p* < 0.0001 comparing with the effect of the same type of particles on HaCat cell line. ### *p* < 0.001 and #### *p* < 0.0001 comparing with the effect of the same type of particles on A375 cell line. Data are presented as mean value ± SD, *n* ≥ 3.

When comparing cell viability for the different formulations at 100 μM in the three cell lines tested, overall, B16F10 was the most affected cell line following incubation with GNPs (Figure 6).

### 3.3. Human Melanoma Xenograft Model

All mice were monitored from 60 to 90 days, depending on the tumor growth rate, and showed normal water and food intake, respiratory activity, posture (independently of the presence or size of the tumor), and mobility, confirming that the animal welfare was not compromised. The weight was registered throughout the study until sacrifice, and no significant (*p* < 0.05) weight variations were observed over time. The group of animals without tumors presented an average weight of 29.0 ± 0.4 g (27.3 ± 0.5 g to 30.5 ± 3.2 g; *n* = 6). By its turn, the animals with tumors showed an average weight of 27.6 ± 0.3 g (21.2 ± 1.6 g to 30.1 ± 0.7 g; *n* = 13). These differences in overall weight were already present before the xenograft induction, during the adaptation period.

The variation of tumors volume and time of appearance after cell inoculation in mice was high, ranging from 5 to 1000 mm^3^, and from two weeks to two months, respectively. Despite this fact, SCID mice were monitored twice a week, during all in vivo experiment, and have shown stable body conditions with a BCS > 3.

No visible micro- or macrometastases of melanoma were found in lungs and there were no size variations of these organs between the control and tested groups. Due to the variability in tumor volumes among the groups, differences between the tumors were only compared within the same animal, before and after treatment.

### 3.4. In Vivo Evaluation of Safety and Efficacy from the Photothermal Therapy Procedure

The degree of erythema was evaluated by colorimetry, analyzing the difference in a* (AU) before and after laser irradiation. A large variation between animals in each group was attained; the control group (without tumor and nanoparticles) showed a mean difference of a* value of 1.2 ± 1.0 up to 2.7 ± 0.4 after 5 and 10 min of laser irradiation, respectively. In presence of the tumor, the difference of the value of a* was very similar among animals after laser irradiation, confirming the safety of the laser source used for healthy and tumoral tissues. In the case of the tumor group dosed with nanoparticles, the difference of a* value increased to 2.8 ± 0.4 and 4.3 ± 1.3 after 5 and 10 min of laser irradiation, respectively. Nanoparticle local administration suggested a hypothetic concentration of the generated heat in the target area.

**Figure 7:**
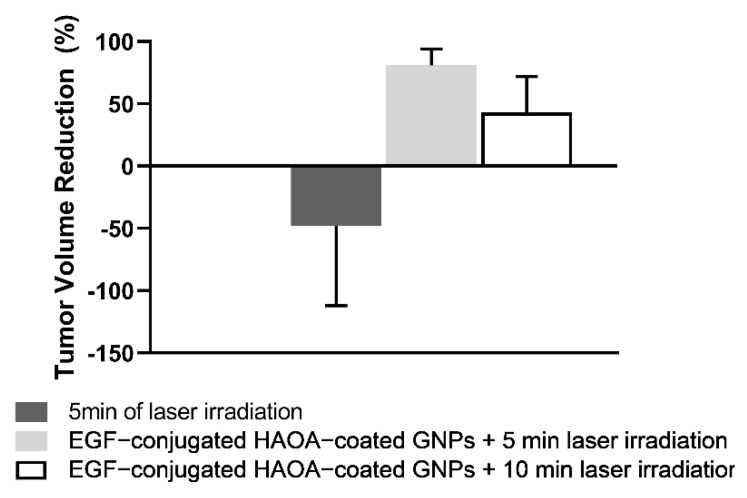
Tumor volume reduction of the different test groups, including control or no treatment, and after respective treatments, namely, 5 min laser irradiation, EGF-conjugated HAOA-coated GNPs + 5 min laser irradiation and EGF-conjugated HAOA-coated GNPs + 10 min laser irradiation. Data are presented as mean value ± SEM, *n* ≥ 3.

After animal euthanasia, the tumors were isolated and measured in order to compare the possible size reduction-effect between the different treatment groups (Figure 7). Although, in the control group, the average tumor size did not change within the 24 h between the application of the treatment and the sacrifice of the animals from the treated groups, mice treated with both nanoparticles and laser showed a reduction on the tumor volume. Mice treated with EGF-conjugated HAOA-coated GNPs and 5 min laser exposure showed an average tumor reduction of 81.1 ± 12.6%. In comparison, when applying the same treatment with nanoparticles, but increasing the laser exposure to 10 min, the average tumor reduction was 43.1 ± 29.4%. For the tumor group exposed only to 5 min laser irradiation, there was no overall change in terms of tumor volume, before and after irradiation.

**Figure 8:**
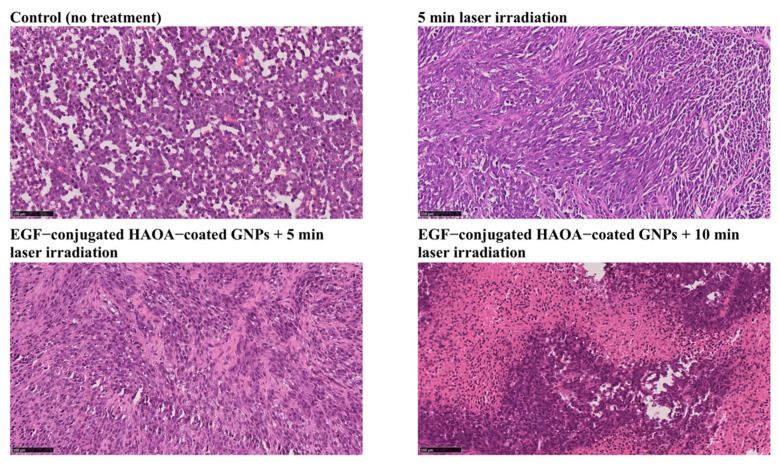
Histological images (H&E staining) of the tumors from the four different groups of animals: control (no treatment), 5 min laser irradiation, EGF-conjugated HAOA-coated GNPs + 5 min laser irradiation and EGF-conjugated HAOA-coated GNPs + 10 min laser irradiation. Scale bar: 100 µm.

Regarding the histological analysis, several necrotic foci were observed in tumor samples from each treated group, in comparison to control group. Tumors exposed to 5 and 10 min laser irradiation combined with EGF-conjugated HAOA-coated GNPs showed signs of coagulative necrosis (Figure 8). On the other hand, on tumors exposed to laser irradiation alone for 5 min (without local injection of nanoparticles), few morphological changes (<30% extended necrosis) were observed.

Both the control and treated groups showed similar morphology and size of the internal organs, namely lungs, kidneys, liver, spleen, and heart. The average tissue indexes for each group included in this study are described in Table 2. Overall, there were no relevant differences in the tissue indexes of the organs between groups.

**Figure 9:**
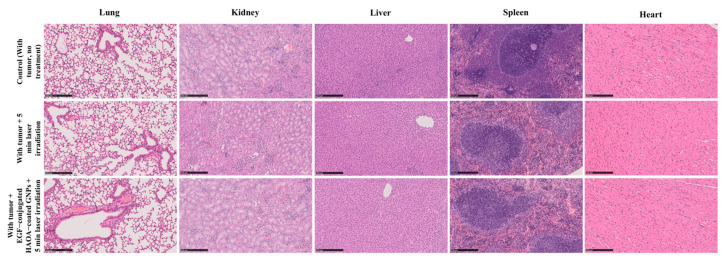
Histological images (H&E staining) of lung, kidney, liver, spleen and heart of three different groups of animals: animals with tumor, no treatment; with tumor + 5 min laser irradiation; and with tumor + EGF-conjugated HAOA-coated GNPs + 5 min laser irradiation. All images are representative of the harvested organs, which showed no inter-group histological alterations. Scale bar: 250 µm.

Attending the histology of the different organs (i.e., heart, kidney, liver, spleen, and lung) no morphological changes were observed when comparing animals from different groups (Figure 9).

## 4. Discussion

This proof-of-concept study for melanoma assessed the in vitro safety of a GNP formulation, as well as the safety of NIR laser irradiation (811 nm) exposure in a SCID hairless mice melanoma model. Additionally, the efficacy of laser irradiation combined with nanoparticles was evaluated for treatment of melanoma in SCID hairless mice. All animals showed no abnormal behavior or change of posture associated to suffering, neither any significant weight loss over time, confirming that the animal welfare was not compromised.

The use of xenograft models are often associated with some variation in tumor growth and evolution [21]. As reported in this study, and also in other publications [57,58], the xenograft model showed different tumor growth rates and, consequently, high variation in tumor size, which extended the follow-up interval (60–90 days). It is hypothesized that high variability depends on distribution of the cells within the tissues after injection, time of vascularization of the injected area, and natural variability between animals, such as mentioned by others [58]. Moreover, it is believed that adaptative and innate immunity of the animals can play a role on xenograft development. For instance, natural killer cells are reported by their limiting action over the growth and establishment of human cells lines in immunodeficient strains, having been associated with delayed tumor growth and increased variability [29]. Due to tumor growth variability, treatments were administered to the animals at different times, when individual tumor volumes required for inclusion in the study where achieved. Despite this, the existence of tumors with different volumes was unavoidable, even within the same treatment group, reinforcing the need to evaluate treatment efficacy through the tumor volume reduction. However, factors such as the tumor volume prior to treatment, the administered dose of the nanoparticles and radiation applied, must be taken into account in this evaluation. Moreover, at the time of treatment the animals had not the same exact age which is a limitation of the xenograft models. Additionally, xenograft models require the use of immunocompromised mice [21,29], which limits the natural mimicking of the immune system. Finally, the animals did not show any lesions in lungs or liver, suggesting that the inoculation of tumor cells successfully led to localized melanoma, without metastasis.

The EGF-conjugated HAOA-coated GNPs used in this study were partly characterized regarding physico-chemical features, in vitro biocompatibility, and cell internalization mechanism in our previous studies [15,35]. The nanoparticles synthesized for this study had similar characteristics, with mean size from 150 to 200 nm, negative surface charge, and spherical morphology. The GNPs size based on the AFM technique also confirmed the results obtained by DLS technique.

To evaluate the potential cytotoxicity of GNP formulations in our study, MTT assays were performed on healthy human keratinocytes (HaCat), human melanoma cells (A375) and murine melanoma cells (B16F10). Uncoated GNPs proved to be safe in all cell lines. A slight reduction on cell viability was observed for the murine melanoma cell line and at the two highest concentrations tested. Nevertheless, cell viability remained always above 80%. On the other hand, and despite preserving cell viability above 70%, when coated and bio-conjugated with EGF, the GNPs showed a higher impact in all cell lines, promoting, in most cases, a reduction of cell viability when compared to uncoated and non-functionalized nanoparticles. A possible cause for this reduction in cell viability may be the fact that the size of HAOA-coated GNPs and EGF-conjugated HAOA-coated GNPs is about double when compared to uncoated GNPs. This greater size may cause a physical “asphyxia” effect in cells, as also reported by others [16,59,60,61]. The precise mechanisms behind cytotoxicity data might be related to differential receptor expression influencing particle internalization, and different sensitivity to physicochemical features of the particles (namely, size and surface charge). The GNPs herein used are coated with HA and further conjugated with EGF, which are ligands for CD44 and EGF receptors, respectively, expressed differently in the cell lines [44,45,46,47]. The presence of the particles in A375 cells was also assessed to support the protocol followed in vivo in terms of time interval separating the GNPs injection and the laser irradiation procedure. A previous work from the group showed the entrance of similar GNPs into the peri-nuclear area of A549 cells 1.5 h after incubation [35]. Thus, herein, it was considered a longer margin for further in vivo application and the internalization was assessed after a period of 4 h of incubation. Once the unbound GNPs were removed prior the analyses, two techniques confirm the presence of GNPs in the cells, which suggests either their internalization or their binding to the surface of the cells.

Afterwards, the safety of NIR laser irradiation was assessed by testing on healthy SCID hairless mice two different exposure times. The dose of gold administered (1.25 mg/kg) took in consideration the in vitro results attained here, in which concentrations of particles up to 80 µM of gold did not show significant toxicity, and this is in agreement with previous findings of the group [15,35]. Of notice, the gold dose used herein was lower than that reported before involving radiation enhancement [62,63]. The radiation dose and time of exposure were selected by taking in consideration irradiation parameters previously reported for photothermal therapy applications [51,52], and exposure limits imposed by law [53,54]. The radiation dose applied was about seven times higher than the exposure limit for a NIR source with the same wavelength. Nevertheless, the exposure limit corresponds to the maximum level of exposure for which no adverse skin effects are expected, but it can be exceeded for diagnostic and therapeutic purposes when the intended benefits of the procedure overcome the potential side effects [54]. Additionally, previous in vitro results from the group [50] were taken into account in this decision. None of the laser irradiation times compromised the skin integrity or promoted signs of injury, either macroscopically or based on histological analysis. The dermis kept its stratified epithelium and its fibrous structure with sweat glands present in the fatty tissue (hypodermis). In a second phase of this in vivo study, the efficacy of NIR laser irradiation alone or combined with the intra-tumoral administration of the EGF-conjugated HAOA-coated GNPs was appraised. To evaluate the degree of erythema induced by laser irradiation on SCID hairless mice, a chromameter was used to compare the color of the skin before and after irradiation. No visible skin burns, nor high degree erythema were observed in any animal. With regard to the data on erythema, it should be highlighted that the tumors developed in the fold-back of the animals’ neck but they grew in different rates, and probably with different deepness. Consequently, it was hypothesized that the vascularity and irrigation of the tumors could have varied, influencing the results obtained in the colorimetry technique.

Overall, in vivo results showed a significant reduction in the volume of melanoma tumor at 24 h-post treatment. The control group did not show any tumor volume changes, contrary to the animals treated both with EGF-conjugated HAOA-coated GNPs and NIR laser using different exposure times, or submitted to laser irradiation during 5 min. The effect of 5 min laser irradiation on tumor volume was not conclusive, since it was tested in a small number of animals, and each animal responded differently to the irradiation. Any increase in the tumor volume could be, for example, a consequence of an inflammatory process induced by the laser irradiation.

The most promising results in this study were observed with the 5 min laser irradiation combined with EGF-conjugated HAOA-coated GNPs, which led to a tumor reduction of approximately 81%. Furthermore, the results observed for the group of animals treated with the 10 min laser irradiation combined with EGF-conjugated HAOA-coated GNPs showed a tumor reduction of around 43%. Several factors may have contributed to this reduction. The GNPs reach their maximum absorption upon laser irradiation with a specific wavelength, promoting local hyperthermia of the tissues. It is described that this local effect can lead to an irreversible cell damage caused by the disruption of cell membrane permeability and protein denaturation [8,9]. Nonetheless, studies on the molecular mechanism behind the antitumoral effect of these multifunctional hybrid nanoparticles are still under way. Moreover, the combination of polymeric coatings and bio-conjugation could probably minimize the interactions of the nanoparticles with macrophages, that range from 0% to 30% in the melanoma tissue [64]. That could lead to a reduction of nanoparticle phagocytosis by these cells, and thus increasing the available intra-tumoral nanoparticle content to ligate to tumoral cells [65].

The safety of EGF-conjugated HAOA-coated GNPs was not yet studied in vivo. As performed by other authors [66], all animals received the same dose of nanoparticles based on their body weight, regardless the tumor volume, despite an intra-tumoral administration has been used. For instance, the animals treated with EGF-conjugated HAOA-coated GNPs in combination with 5 min laser irradiation presented an average volume prior treatment of about forty times smaller than the group of animals treated with the same system for 10 min. This difference in tumor volume between the two groups may be one of the explanations for the lower reduction in tumor volume in the case of treatment of GNPs followed by laser irradiation for 10 min (43% vs. 81%). This comparison of results suggests that other existing variables, not yet studied, might influence the evaluation of the treatment effect based only on tumor volume reduction. Examples of these variables include: tumor physiology; tumor stage (i.e., as the tumor stage advances, the prognosis gets worse and the tumor becomes more difficult to treat); and the nanoparticle dose adjustment based on the tumor volume [67]. Lastly, if the photothermal effect is enhanced by the nanoparticles targeted effect to the melanoma cells and if the dose of the nanoparticles is not proportional to the tumor volume, then tumors with different volumes will not be exposed to a proportional amount of nanoparticles, and thus affecting the extent of the hyperthermia.

In terms of histological analysis, tumors that received nanoparticles and were exposed to NIR laser showed coagulative necrosis, in contrast with the tumors not treated. The histological analysis of tumoral samples from the groups receiving GNPs did not show the presence of GNPs, despite a quantity of particles administered in situ, which might be related with the resolution of the optical microscope and the contrast of the images upon the staining of the samples. Further studies should clarify particle distribution in tumor samples. The tumoral samples from treated animals had a pale H&E staining, when compared to non-treated animals, which may indicate a loss of tumoral cytoplasmatic and nuclear content [68]. Tumors treated only with laser irradiation for 5 min showed histological changes, namely necrosis (<30%). The coagulative necrosis observed in the treated tumors could also result from the denaturation of the tissue structural proteins caused by the photothermal effect, as previously reported by other studies [69,70]. These results were observed 24 h after NIR treatment, which provides information about the immediate treatment efficacy, in accordance to that reported by others [70]. Nevertheless, further studies with longer periods of monitoring after the treatment must be carried out to evaluate other parameters such as particle biodistribution, potential cytotoxicity, and inflammatory and immune responses, among others.

Finally, to evaluate and compare the toxicity of the methodologies tested, tissue indexes of the multiple organs collected and their histological results were examined. Data on tissue indexes showed no significant differences for individual organs among animals without and with tumors, neither among animals receiving different treatments. Similar results are supported by the histological analysis of the multiple organs, which showed no abnormal morphology of the tissues, regardless of the test group in which the animal was included. The use of EGF-conjugated HAOA-coated GNPs proved to highly enhance the photothermal effect in vivo, without affecting the normal function of the organs nor causing any inflammatory systemic response in the 24 h period after nanoparticles administration.

## 5. Conclusions

In conclusion, this proof-of-concept study suggests that the multifunctional hybrid nanoparticle system characterized herein can be a promising new therapeutic strategy for localized melanoma. This therapeutic approach did not show evidence of causing damage to normal skin structure, which indicates the nanoparticle system selectivity towards the melanoma tissues, although further research is necessary to clarify the molecular mechanism underpinning this system. This study also unveils the potential of a unique multifunctional hybrid system with different targeting moieties to approach other localized superficial tumors, such as squamous cell carcinoma, mammary gland tumor, among others.

## Figures and Tables

**Table 1:** Mean size, polydispersity index (PdI) and zeta potential of uncoated, HAOA-coated and EGF-conjugated HAOA-coated GNPs. Data are presented as mean value ± SD, *n* = 3.

GNP Formulation	Mean Size (nm)	PdI	Zeta Potential (mV)
Uncoated GNPs	64 ± 1	0.434 ± 0.007	−24 ± 2
HAOA-coated GNPs	150 ± 2	0.434 ± 0.014	−40 ± 1
EGF-conjugated HAOA-coated GNPs	157 ± 5	0.383 ± 0.046	−19 ± 9

**Table 2:** Average tissue indexes of male hairless severe combined immune-deficient (SCID) mice for each treatment group. Data are presented as mean value ± SEM, *n* ≥ 3.

Group of mice	Tissue Index				
	Lung	kidney	Liver	Spleen	Heart
Without tumor + 5 min laser irradiation	8.1 ± 0.2	13.4 ± 0.1	24.1 ± 0.1	3.8 ± 0.1	7.5 ± 0.1
Without tumor + 10 min laser irradiation	9.0 ± 0.1	14.0 ± 0.1	25.0 ± 1.2	3.7 ± 0.6	8.3 ± 0.3
Control (With tumor no Treatment)	8.8 ± 0.8	13.2 ± 1.1	24.4 ± 1.8	4.6 ± 0.2	8.0 ± 0.5
With tumor + 5 min laser irradiation	8.5 ± 0.3	13.3 ± 0.4	23.3 ± 0.6	4.0 ± 0.3	8.0 ± 0.3
With tumor + EGF-conjugated HAOA-coated GNPs + 5 min laser irradiation	7.9 ± 0.1	13.1 ± 0.1	25.9 ± 0.6	4.1 ± 0.2	6.6 ± 0.1
With tumor + EGF-conjugated HAOA-coated GNPs + 10 min laser irradiation	7.7 ± 0.5	13.5 ± 0.6	24.7 ± 0.9	4.8 ± 0.2	7.5 ± 0.3

## Data Availability

The databases created and analyzed throughout the study are available upon request to the corresponding author.

## Supplementary Materials

The following are available online at https://www.mdpi.com/2218-273X/11/4/511/s1, Figure S1: AFM height images of A375 cells alone (control) and after 4 h of incubation with different GNP formulations. Additional height and peak force error images showed with more detail cells incubated with HAOA−coated GNPs leading to hypothesize the presence of GNPs aggregates in the surface (arrow) as well as inside of the cell (circle), Figure S2: Representative images of A375 cells alone (control) and after 4 h of incubation with different GNP formulations at 40× magnification. The GNPs appear highly aggregated as darker structures, which seem to be in the surface (arrow) as well as inside of the cell (circle). Scale bar, 75 µm.
