# Supplementary material for: Proof-of-Concept Study of Multifunctional Hybrid Nanoparticle System Combined with NIR Laser Irradiation for the Treatment of Melanoma
Source: Biomolecules. 2021 Mar 30;11(4):511. doi: 10.3390/biom11040511 (PMC8103244; doi:10.3390/biom11040511)
Supplement: Supplementary file 1 [file biomolecules-11-00511-s001.pdf]

biomolecules

MDPI

Supplementary Material

Proof-of-Concept Study of Multifunctional Hybrid Nanoparticle System Combined with NIR Laser Irradiation for the Treatment of Melanoma

Joana Lopes [1,†], Tânia Ferreira-Gonçalves [1,†], Isabel V. Figueiredo [2,3], Cecília M. P. Rodrigues [1], Hugo Ferreira [4], David Ferreira [5], Ana S. Viana [6], Pedro Faísca [7,8], Maria Manuela Gaspar [1], João M. P. Coelho [4], Catarina Oliveira Silva [1,9,‡] and Catarina Pinto Reis [1,4,‡,*]

1. Research Institute for Medicines (iMed.ULisboa), Faculty of Pharmacy, Universidade de Lisboa, Av. Professor Gama Pinto, 1649-003 Lisboa, Portugal; joanamargaridalopes@campus.ul.pt (J.L.); taniag1@ff.ulisboa.pt (T.F.-G.); cmprodrigues@ff.ulisboa.pt (C.M.P.R.); mgaspar@ff.ulisboa.pt (M.M.G.); catarina.m.oliveira.silva@gmail.com (C.O.S.)
2. Pharmacology and Pharmaceutical Care Laboratory, Faculty of Pharmacy, University of Coimbra, Azinhaga de Santa Comba, 3000-548 Coimbra, Portugal; isabel.vitoria@netcabo.pt
3. Institute for Clinical and Biomedical Research (iCBR), Faculty of Medicine, University of Coimbra, Azinhaga de Santa Comba, 3000-548 Coimbra, Portugal
4. Instituto de Biofísica e Engenharia Biomédica, Faculdade de Ciências, Universidade de Lisboa, Campo Grande, 1749-016 Lisboa, Portugal; hugoferreira@campus.ul.pt (H.F.); jmcoelho@fc.ul.pt (J.M.P.C.)
5. MED—Mediterranean Institute for Agriculture, Environment and Development. Department of Veterinary Medicine, University of Évora, Pólo da Mitra, 7002-554 Évora, Portugal; ferreiradav@gmail.com
6. Centro de Química Estrutural, Faculdade de Ciências, Universidade de Lisboa, 1749-016 Lisboa, Portugal; apsemedo@fc.ul.pt
7. CBIOS—Research Center for Biosciences & Health Technologies, Universidade Lusófona de Humanidades e Tecnologias, Campo Grande 376, 1749-024 Lisboa, Portugal; pedrofaisca76@gmail.com
8. Faculty of Veterinary Medicine, Universidade Lusófona de Humanidades e Tecnologias, Campo Grande 376, 1749-024 Lisboa, Portugal
9. Department of Biomedical Sciences, Faculty of Pharmacy, University of Alcalá, Ctra. A2 km 33,600, Campus Universitario, 28871 Alcalá de Henares, Spain

* Correspondence: catarinareis@ff.ulisboa.pt; Tel.: +351-217-946-429 (ext. 14244); Fax: +351-217-946-470

† These authors contributed equally to this work.

‡ These authors share senior authorship.

1. A375 cells handling for optical and atomic force microscopy analyses

Human melanoma cells (A375) were grown in DMEM with glucose (4,500 mg/L), supplemented with 10% FBS and 100 IU/mL penicillin and 100 μg/mL streptomycin (Invitrogen; complete medium). Cells were preserved at 37 °C under a 5% CO_2 atmosphere and cultures were checked and maintained every 2-3 days, until achieving a confluence of about 80%. Succinctly, cells were seeded in 96-well plates (200 μL) and 24-well plates (1 mL) for optical microscopy and AFM analyses, respectively, at 5×10^4 cells/mL and allowed to adhere, overnight, in the culture conditions specified in the main document. Thereafter, the complete medium was removed, and the cells were incubated at 37°C under a 5% CO_2 atmosphere for 4 h with GNPs. After the incubation period, medium was discarded, and fresh medium was added. Cells were analyzed by both techniques.

2. Atomic force microscopy analysis

The methodology followed are described in the material and methods section of the main document, with the difference that these samples with cells were acquired in small glass slides instead of a freshly cleaved mica.

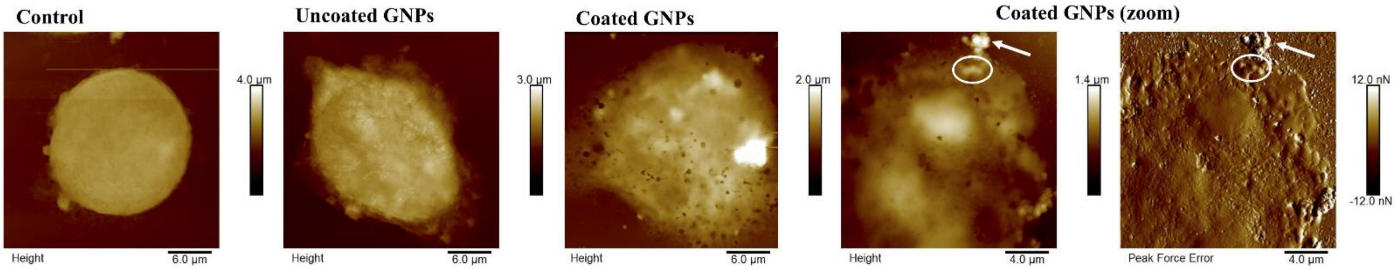

Figure S1. AFM height images of A375 cells alone (control) and after 4 h of incubation with different GNP formulations. Additional height and peak force error images showed with more detail cells incubated with HAOA-coated GNPs leading to hypothesize the presence of GNPs aggregates in the surface (arrow) as well as inside of the cell (circle).

3. Optical microscopy analysis

Brightfield optical microscopy images were acquired with Invitrogen EVOS™ FL Auto2 imaging system (Invitrogen, Thermo Fisher Scientific).

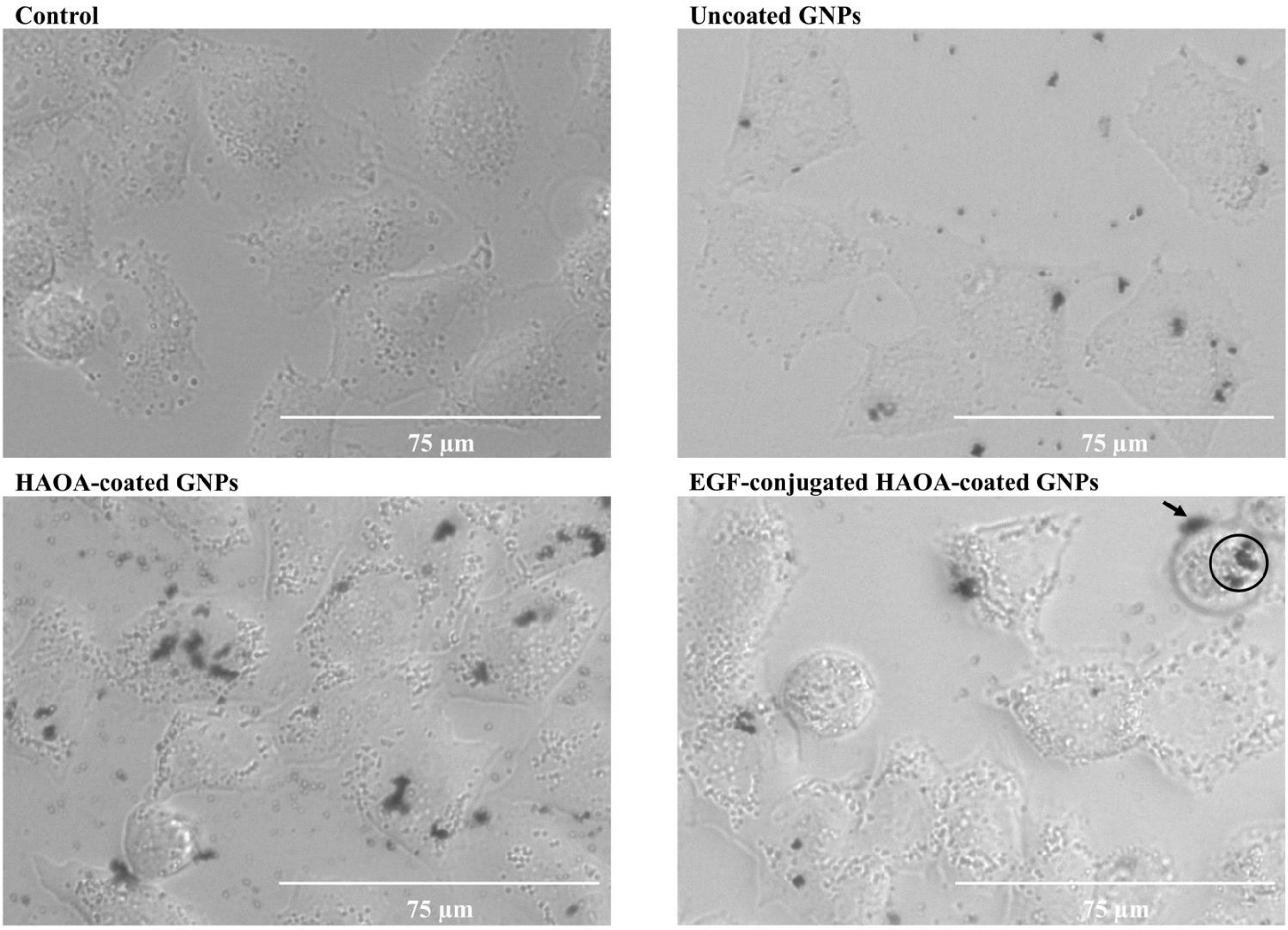

Figure S2. Representative images of A375 cells alone (control) and after 4 h of incubation with different GNP formulations at 40x magnification. The GNPs appear highly aggregated as darker structures, which seem to be in the surface (arrow) as well as inside of the cell (circle). Scale bar, 75 µm.
